# Reasons for Delayed Diagnosis of Pediatric Acute Appendicitis during the COVID-19 Era: A Narrative Review

**DOI:** 10.3390/diagnostics13152571

**Published:** 2023-08-02

**Authors:** George Pantalos, Smaragda Papachristidou, Eleftheria Mavrigiannaki, Nikolaos Zavras, George Vaos

**Affiliations:** 1 Pediatric Intensive Care Unit, Penteli General Children’s Hospital, 15236 Athens, Greece; 2 Second Department of Pediatrics, School of Medicine, National and Kapodistrian University of Athens, P. & A. Kyriakou Children’s Hospital, 11527 Athens, Greece; smagia.gsp@gmail.com; 3 Department of Pediatric Surgery, School of Medicine, National and Kapodistrian University of Athens, “Attikon” General University Hospital, 12462 Athens, Greece; ele17mav@yahoo.gr (E.M.); gvaos@med.uoa.gr (G.V.)

**Keywords:** COVID-19, appendicitis, diagnosis, delay, pediatric surgery

## Abstract

Global pandemics cause health system disruptions. The inadvertent disruption in surgical emergency care during the Coronavirus Disease 2019 (COVID-19) pandemic has been the topic of several published studies. Our aim was to summarize the reasons that led to the delayed diagnosis of pediatric appendicitis during the COVID-19 era. This systematic literature search evaluated studies containing pediatric appendicitis patient data regarding outcomes, times to hospital admission or times from symptom onset to emergency department visit. Studies elucidating reasons for delays in the management of pediatric appendicitis were also reviewed. Ultimately, 42 studies were included. Several reasons for delayed diagnosis are analyzed such as changes to public health measures, fear of exposure to COVID-19, increased use of telemedicine, COVID-19 infection with concurrent acute appendicitis, recurrence of appendicitis after non-operative management and increased time to intraoperative diagnosis. Time to hospital admission in conjunction with patient outcomes was extracted and analyzed as an indicative measure of delayed management. Delayed diagnosis of acute appendicitis has been documented in many studies with various effects on outcomes. Suspicion of pediatric acute appendicitis must always lead to prompt medical examination, regardless of pandemic status. Telemedicine can be valuable if properly applied. Data from this era can guide future health system policies.

## 1. Introduction

The Coronavirus Disease 2019 (COVID-19) worldwide pandemic presented a serious challenge with regard to health systems around the globe. Infection by severe acute respiratory syndrome coronavirus 2 (SARS-CoV-2) and the natural course of the disease were initially unknown. Institutions and governments hastily adopted groundbreaking policies to prevent the spread of the virus such as sealing country borders and enforcing lockdowns (or “stay-at-home” policies) [1]. These altered many facets of daily life including medicine. The fear and uncertainty of the public, along with a multitude of other factors, contributed to the postponement of regular medical examinations or procedures. Such delays and difficulties in access also applied to emergency department (ED) visits, with a sharp decline in emergency non-COVID-19 cases noted during the lockdown period [2,3,4]. The burden on healthcare systems attributed to COVID-19, especially in densely populated areas, highlighted the need for significant changes in the management of emergency non-infectious surgical diseases. The aforementioned changes varied across countries, cities and institutions. The reluctance shown by both patients and their families to visit EDs, as well as the hurried clinical evaluations by medical staff, led to the delayed diagnosis and treatment of other common diseases, which resulted in severe manifestations that could otherwise have been avoided [5,6,7,8].

Pediatric appendicitis is one of the most common pediatric surgical emergencies. In general, appendicitis as a disease, in both adults and children, has been well documented [9]. Emphasis has always been placed on prompt diagnosis so that optimal outcomes with lower rates of complication can be achieved. However, the accurate diagnosis of appendicitis can be difficult and remains a challenge in pediatrics, especially in cases involving younger patients. Delays in time from the presentation of symptoms to surgery have linear associations with complications including perforation, peritonitis, sepsis and overall increased morbidity [9,10]. Younger patient age also correlates with perforation risk [10]. There is consensus among authors on the natural history of appendicitis although some authors have hinted at appendicitis not necessarily being a progressive disease [9,11].

While some recent meta-analyses and systematic reviews [11,12] have assessed the impact of COVID-19 on various pediatric appendicitis characteristics and outcomes, thorough discussion on qualitative matters is essential, as high heterogeneity with conflicting results can be noted among studies. This narrative review of the available literature aims to highlight and analyze the reasons leading to the delayed diagnosis of pediatric appendicitis during the COVID-19 era. Another aim of this study is to present an overview of relevant literature on this topic. Delays in diagnosis and relevant outcomes such as complication rates, morbidity, mortality, imaging use, safety and the cost-effectiveness of non-operative management (NOM) are also matters of discussion. Furthermore, the importance of extracting and understanding this information to help guide current and future practice regarding pediatric appendicitis management must not be underestimated. Valid reasons that remain after the pandemic can then be mitigated across different health systems and countries to minimize diagnostic delays for pediatric appendicitis.

## 2. Materials and Methods

**Figure 1:**
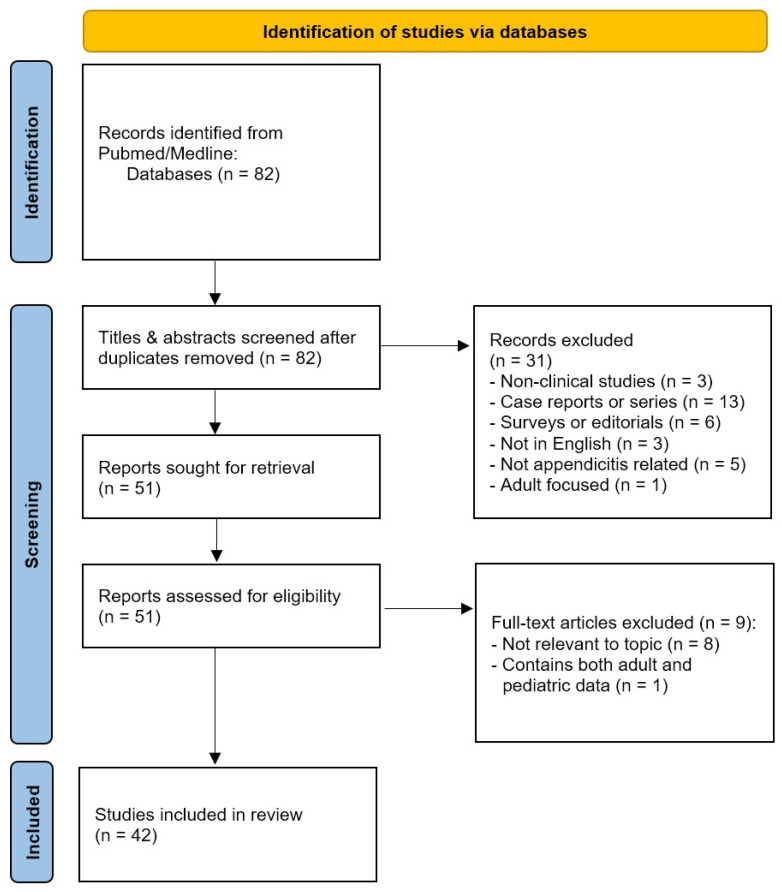
PRISMA 2020 flow diagram for the records identified after searching the databases.

A systematic literature search in Pubmed and Medline for relevant articles was performed until 1 May 2023 (Figure 1). Search terms included “delay”, “time”, “COVID-19”, “pediatric” and “appendicitis” (Appendix A). Original prospective or retrospective cohort studies, randomized controlled trials and systematic reviews were included. We focused on articles reporting on pediatric patients (age < 18 years) diagnosed with appendicitis during the pandemic period that contained patient data regarding outcomes and, ideally, times to hospital admission or times from symptom onset to medical examination or to operative management. Publications were screened by reviewing their abstracts. Subsequently, the full-text evaluation of articles for relevance to the topic was completed. The reference lists of the selected studies were also searched. Studies were reviewed by two authors (G.P. and S.P.) while content experts (G.V. and N.Z.) ensured that the most pertinent studies on the subject matter had been included. Adult, non-English, non-clinical studies and editorials, abstracts or expert opinions were excluded from this review.

## 3. Results

**Figure 2:**
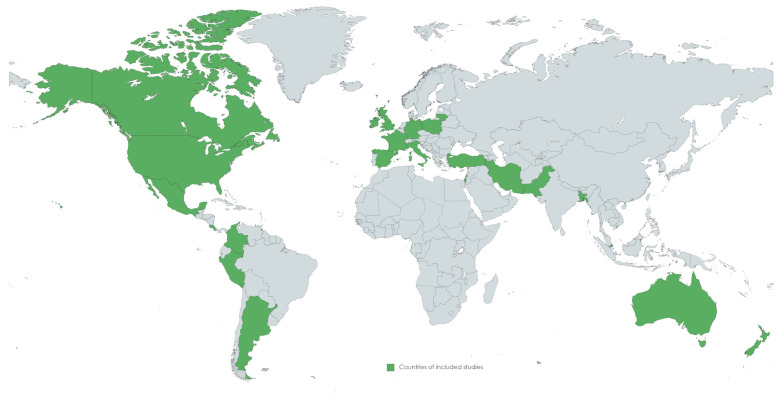
Countries of origin of included studies.

A total of 82 studies were identified after the initial search, 51 studies of which remained for full-text evaluation after abstract screening and deduplication. After full-text evaluation, 42 studies were included. The selected articles involved 2 systematic reviews/meta-analyses, 33 retrospective studies, 6 prospective cohort studies and 1 case-control study. Their quality was deemed sufficient for the purposes of this review. One case series study with a retrospective cohort component was included because it clarified some of the reasons for delays [13]. A narrative review was then conducted focusing on reasons for delays in the diagnosis of pediatric appendicitis during the COVID-19 era in accordance with the Preferred Reporting Items for Systematic Review and Meta-analysis (PRISMA) guidelines [14]. Key findings including reported outcomes from the included studies are presented in the following section. The publication details of the included studies are presented in Table 1 and Figure 2.

## 4. Discussion

### 4.1. Key Findings

An assortment of reasons delaying the diagnosis of pediatric appendicitis were discerned after the thorough appraisal of the literature (Table 2). Each cause will be discussed separately although it must be noted that there is a significant overlap between them. Time to hospital admission, duration of symptoms and hospitalization time until surgery will be discussed further as indicative measures of delayed diagnosis. Patient outcomes as conveyed by selected studies will be cited in relation to time to hospital admission so that the reader can appraise their potential importance.

### 4.2. Reasons for Delayed Diagnosis

#### 4.2.1. Public Health and Social Measures (PHSMs)

After the initial outbreak in December 2019 and the spread of the SARS-CoV-2 virus from China to the rest of the world, efforts were made to limit the spread of COVID-19 [52]. Governments responded by implementing a series of lockdowns or stay-at-home orders and enforced travel restrictions between areas, cities, regions and countries. The stringency of social distancing or isolation varied across nations as referenced by numerous studies [20,27,53]. Stricter policies could, however, amplify fear and uncertainty, thus delaying presentation to the hospital as was potentially the case for New Zealand vs. Australia [20]. The World Health Organization has completed a database project to collect and archive PHSMs as implemented by individuals, institutions, communities, local and national governments and international bodies to limit the spread of COVID-19 [1]. These policies focused on several aspects to slow the spread of COVID-19 [54] but contributed to perceived significant delays in the diagnosis of common emergency conditions like appendicitis.

Healthcare resource limitations were deemed necessary in order to allocate as many beds, staff, etc. as possible to the treatment of COVID-19 patients. Many surgical and medical departments were converted to COVID-19 wards and even operating rooms (ORs) were transformed into makeshift I.C.Us. [55]. Elective surgeries were postponed. This also meant physicians and nurses could be assigned from surgical to infectious departments during peak periods. These strategies were devised and executed to conserve health system resources and prevent hospitals from being overwhelmed by the surge in COVID-19 cases, which could lead to unacceptably high mortality [3]. The peak of the first wave of COVID-19 during March 2020 was arguably the most overwhelming for health systems in several countries [56]. The shift to the NOM of appendicitis as a strategy to spare hospital resources in cities stricken by the pandemic must also be mentioned.

Travel restrictions between cities, provinces and regions and the assignment of pediatric hospitals or wards to adult COVID-19 care could have delayed pediatric appendicitis diagnosis by lengthening the times to examination by a surgeon and admission to a hospital [27]. Studies focusing on rural area populations have illustrated such delays during pre-pandemic periods [57]. Curfews and fines for their violation also constitute reasons for delays.

Pediatric appendicitis outcomes can also be affected by lower socio-economic status as a consequence of hindered access to healthcare. This was studied before the pandemic [28,57,58,59,60]. The associations between pediatric appendicitis outcomes and lower socio-economic status groups and poor nations that were highly affected by the pandemic warrant further study as there is no relevant evidence to date.

The restriction of healthcare access may have had the opposite intended effect regarding resource deployment given that increases in complicated case numbers, length of stay (LOSs), operation durations and numbers of admissions and readmissions were recorded [3,13,33,37,39]. Higher ratios of complicated pediatric appendicitis were demonstrated in countries that were hit harder by the pandemic including the USA, Germany and other countries where uncomplicated appendicitis case volume dropped [7,8,38,61,62,63], but this was not always the case [24]. This effect was mostly associated with the first peak period of COVID-19 and was later reversed [63].

The importance of this secondary effect of the pandemic, namely the hampered access to specialized pediatric care that could delay diagnosis, must not be underestimated. Taking into consideration the data from most of the included studies, we strongly recommend that all children with symptoms suggestive of surgical emergencies must be examined and accurately diagnosed if optimal outcomes with minimal complications are to be achieved [20,27]. The provision of healthcare pathways for medical examination is vital, even during a pandemic [64]. While predictions of potential health crises in the future cannot be made, there is a wide consensus that preparing strategies for policy making and staff and resource allocation with current knowledge is imperative [33,65].

#### 4.2.2. Fear of Exposure by Parents/Caregivers and/or Patients

The number of ED visits decreased worldwide as parents were consumed with the fear of exposing children to SARS-CoV-2 [2,3,4]. A multitude of studies in Italy, Spain, the U.S.A., the U.K., Canada and France have stated that parents delayed hospital visits out of fear. The same studies demonstrated worse outcomes, higher complication rates and LOSs, in particular for pediatric patients treated during the first wave of COVID-19 [2,3,13,15,19,27,37,46]. Parental fear could have been exacerbated by being in countries with higher COVID-19 alert levels. In other studies, parents did not hesitate to bring their children to EDs, although these studies reported an increase in the percentage of patients presenting at EDs at least 48 h following symptom onset [31]. The lack of clear public health guidance on presenting to EDs for severe symptoms could confuse parents and delay the assessment of their children [20]. Parental anxiety and the psychosocial impact of COVID-19 on society as a whole, termed Coronaphobia, have been analyzed further by Dubey et al. [66]. Level of family education level did not correlate with delayed visits to hospitals [28,66].

The fear experienced by children, secondary to parents’ anxiety regarding the ongoing pandemic, could explain their hesitation to mention their initial symptoms. The recognition of symptoms by parents could also be hampered by their subjective nature when parents actively tried to avoid healthcare or ED visits [4].

Van Amstel et al. remark on the gradual removal of lockdown measures after the first months of the pandemic, which led to less fear and a lower threshold for seeking medical care even for milder symptoms [63]. Öztaş et al. mention that their results showed no difference in the overall frequency of appendicitis during the first year of the pandemic [47]. They attributed this finding to the fact that the included parents thought that children were not as likely to be infected by COVID-19 in general and that pediatric emergency departments were viewed as safer in that regard. Hence, there was no decrease in hospital visits [47].

#### 4.2.3. Fear of Exposure by Medical Staff

The diagnosis of pediatric appendicitis can be delayed by numerous factors including, but not limited to, inconsistency in clinical presentation, varying gravity of symptoms, and difficulty in communication and the clinical examination of children, especially younger children [67,68]. Many authors have identified the deleterious effect that fear of exposure by medical professionals had in multiplying these already established difficulties. It has been mentioned that an emergency doctor’s fear of contact with patients can lead to haste in the examination of a patient, the cornerstone of diagnosis; this has been commented on by some authors [13,47]. Snapiri et al. allude to potential misdiagnoses caused by rushed examination, telemedicine use and reluctance to instruct patients to visit EDs. These factors led to serious clinical deterioration and complications for their reported cases [13].

The skyrocketing demand for and ineffective distribution of Personal Protective Equipment while the first wave peaked contributed to fear of contamination by staff and delays for many components of effective healthcare. Meanwhile, concern for staff protection from exposure to SARS-CoV-2 indicated that avoiding or delaying surgery could protect surgery personnel by limiting their exposure to possibly contaminated body fluids or generated aerosols from patient tissues [32,69,70].

A reduction in the volumes of diagnostic imaging modalities such as ultrasound or computed tomography scans in general during the first months of the pandemic has been clearly demonstrated [29,71,72]. However, we can assume that delays in imaging could have led to the delayed diagnosis of pediatric appendicitis given the increase in diagnostic imaging use for suspected appendicitis during the pandemic as reported by some authors [43]. As Kim et al. point out, the positive imaging pediatric appendicitis case numbers were similar to those of previous years, and Horst et al. noted that the numbers of patients treated surgically were also similar [29,71]. These findings could be attributed either to pediatric patients presenting in a timely fashion to EDs as before or to having pediatric cases routed to dedicated pediatric hospitals and thus inflating these numbers [29,71].

#### 4.2.4. Telemedicine Use 

Telemedicine use became more widespread during the pandemic, mostly via phone or video calls [73]. Telemedicine is at the intersection where the previous three reasons meet. Fear of infection by healthcare professionals, fear experienced by parents and public health policy all promoted its use. Telemedicine evaluation is inherently incomplete because examination is unfeasible, and so, management could be varied. Where the main aim of a consultation is to promote home care, e.g., with antibiotics, delays in the diagnosis of appendicitis could occur [74]. Video use could possibly help in recognizing critically ill children [13]. An interesting side effect of greater telemedicine use could be the resolution of some cases of simple appendicitis, either spontaneously or with oral antibiotic therapy, due to its being essentially non-operatively managed at home with a presumed diagnosis. This has been suggested by Tankel et al. [75].

#### 4.2.5. COVID-19-Positive Pediatric Patients with Suspected Appendicitis

SARS-CoV-2 infection has not been commonly associated with causing pediatric appendicitis [76] although such a connection was initially hypothesized [77]. It is necessary to point out that while research had to validate this causative relationship, the management of patients was ongoing. 

Potential perioperative morbidity in COVID-19-positive children with suspicion of appendicitis was an initial concern, and so, surgery was delayed to prevent perioperative pulmonary complications. This line of thinking was supported by adult-population experience as originally reported by the Lancet’s “COVIDSurg” Collaborative [32,78]. This was not later confirmed by the same author for the pediatric population during the pandemic [79]. Similar perioperative safety for infected children wasdemonstrated by other authors, making these delays for surgery inappropriate [32,79,80,81,82].

Multisystem inflammatory syndrome in children (MIS-C) comprises a dangerous clinical entity with multiple-system involvement and possible multiorgan damage or failure [83]. It can manifest with a multitude of symptoms including but not limited to a rash, fever, abdominal pain, diarrhea, vomiting, conjunctivitis and shock. Differential diagnosis from Kawasaki disease and toxic shock syndrome can be challenging [84]. Prior infection or contact with a positive SARS-CoV-2 case and lack of an alternative diagnosis are among the criteria for establishing a diagnosis of MIS-C [85]. MIS-C abdominal pain can be severe and can mimic appendicitis [84,86,87]. MIS-C can also manifest with severe gastrointestinal diagnoses including appendicitis [88]. Increased clinical suspicion of MIS-C could have delayed diagnosis of appendicitis and vice versa [34,89,90]. Avoiding surgery for MIS-C and administering corticosteroid therapy while expediting surgery for complicated appendicitis is rational, given their respective complications [34,91,92].

#### 4.2.6. Recurrent Appendicitis after Non-Operative Management

Delayed diagnosis might occur as recurrent appendicitis could be underappreciated by physicians not familiar with NOM and its complication rates, namely high failure or early recurrence and late recurrence after NOM [93]. NOM was sparingly employed for pediatric appendicitis cases before the pandemic. The novel application of NOM during the initial pandemic assault has shown that NOM can be an effective management strategy [11,94,95]. When proper standards and strict patient selection criteria for applying NOM are set, informed decision making by parents can be made with acceptable cost effectiveness [9,35,42].

#### 4.2.7. Delays in Primary Operative Management or Increased Time to Operating Room

Given that appendicitis poses a diagnostic challenge, consideration must be given to aspects of delays in primary operative management during the pandemic since the diagnosis of appendicitis is ultimately verified intraoperatively, predominantly for equivocal cases [9]. It is possible that during the pandemic, more effort was made to establish a correct diagnosis preoperatively. This could have been due to greater imaging use, more direct senior consultant involvement and a higher threshold for surgical exploration [43,45]. Cases that were diagnostically uncertain were more likely to be managed non-operatively during the pandemic, as recommended by guidelines [96,97]. This led to two outcomes: higher numbers of false-positive appendicitis cases treated by NOM and lower negative appendicectomy rates [43]. Conversely, lower negative appendicectomy rates can be associated with either treating patients more easily with NOM or better diagnostic accuracy. However, improving diagnostic accuracy includes serial imaging, laboratory results and clinical examinations after an observation period; hence, inherent delays in this process can be assumed [9,49,92]. On a side note, achieving lower negative appendicectomy rates is interesting because it conserves resources and protects children from risks inherent to surgery [43,98].

Surgery was delayed during the first wave of COVID-19 due to knowledge gaps regarding the course of the disease. Aerosol generation by diathermy/electrosurgery and laparoscopy had to be eliminated as possible vectors of viral transmission so as to safeguard surgery staff safety [40]. This was suggested by the Royal College of Surgeons of England [97] but not by the Royal Australasian College of Surgeons [99] or the American College of Surgeons [93].

As stated earlier, there was uncertainty regarding whether operating on COVID-19-positive children with suspected appendicitis could increase perioperative pulmonary and immune-system morbidity. This may have contributed to delays in surgery and thus diagnosis.

Reference must be made to SARS-CoV-2 PCR testing or rapid antigen tests. At the outset of the pandemic, these tests were either unavailable, costly or had long waiting times for result retrieval. There is evidence that SARS-CoV-2 testing delayed appendicectomies during the pandemic [51]. As the pandemic continued, all these factors improved; however, wide testing meant that most surgeons waited for SARS-CoV-2 PCR patient results before operating. The study by Ergün et al. established with statistical significance that time from hospital admission to surgery or NOM was indeed greater during the pandemic but did not lead to increased perforation rates for children [41]. This has been the case for adults [9,92]. Consequently, waiting for PCR results is cost-effective and safe in general. We can therefore surmise that some—mainly equivocal—cases, treated and diagnosed by primary surgical management, could have faced delays caused by hospital logistics and appendicitis management changes.

### 4.3. Time to Hospital Admission, Duration of Symptoms and In-Hospital Delays

Duration of symptoms until hospital presentation can be analyzed as a criterion associated with delayed diagnosis. Delayed time to presentation and hospital admission is invariably linked to delayed diagnosis. Researchers have indicated that perforation often occurs prior to hospital admission, and delays in time to admission increase the risk among the adult population. Notably, time to OR or in-hospital delays after admission do not seem to affect perforation rates or outcomes [92,100]. Additionally, some authors have hypothesized that perforated vs. simple appendicitis can occur due to different biological processes wherein perforation occurs rapidly in complicated cases without it being a strictly time-dependent effect [92,101].

Pediatric-population research has proposed that for every hour post ED triage until surgery, there is a 2% increase in the odds of perforation [5]. The perforation of the appendix occurs in 20% of children after 36–48 h from symptom onset, with rates increasing further after 48 h [102,103]. The systematic review and meta-analysis by Motazedian et al. has revealed that the perforated pediatric appendicitis rate has significantly increased post-pandemic in comparison to the pre-pandemic period [23].

Several retrospective cohort studies included in this study compared COVID-19-era pediatric appendicitis patients with control groups from previous years. Some of these articles reported times to admission or durations of symptoms and associated outcomes like complicated appendicitis odds, LOSs, complication rates, imaging use, negative appendicectomy rates and readmission or reintervention rates. These outcomes are presented, in relation to similar or increased times to hospital admission as reported in the included studies, in the form of a narrative review. Due to the high heterogeneity among the studies, no assumption can be made about causative relationships between the outcomes and the times to hospital admission.

Indeed, certain recent articles reveal similar times to presentation during the pandemic. Quaglietta et al. conducted a study that found odds of complicated appendicitis increased by 81% for their COVID-19 patient cohort (in the period from February 2020 to June 2021 vs. the same period in 2018–2019). Comparable durations of symptoms at presentation to a hospital were noted without reaching statistical significance. A difference of one day between presentations was associated with odds of perforation that were increased by 26%. The COVID-19 complicated-appendicitis subgroup needed to travel a greater distance to reach surgical care facilities as government policy instructed patients to reach specific specialized pediatric centers [27]. Similarly, some authors showed similar times from onset of symptoms to hospital admission, complication rates and complicated appendicitis rates between pandemic and control cohorts [21,22,36,40,44,47]. Moreover, the Anzscraft Collaborative, consisting of six centers in Australia and New Zealand, studied how the presumed delayed presentation of pediatric appendicitis during COVID-19 could influence the severity of the disease. They found no differences in the durations of symptoms, sepsis and complicated disease and complication rates. Interestingly, New Zealand patients reported a longer duration of symptoms at presentation for the COVID-19-era cohort (2.7 vs. 1.7 days) and higher rates of complicated disease (44.7% vs. 30.2%). These finding, though not statistically significant, could be due to the unified and stricter New Zealand government policy as opposed to that of Australia [20]. La Pergola et al. found no significant differences in terms of days from symptom onset to hospital admission, not even between the first and second month of the pandemic period. Furthermore, the prevalence of appendicitis and numbers of complicated appendicitis cases were equivalent between cohorts. Their results did not achieve statistical significance [24]. Montalva et al. reported that the mean duration of symptoms was two days in both the pandemic and pre-pandemic periods. The same was true for delayed presentation after three days. Additionally, fewer pandemic-cohort patients consulted with primary care physicians whereas the numbers of complicated appendicitis cases, LOSs and complication rates showed no differences [46]. Comparable results were reported by Del Giorgio et al. [50]. Some studies identified a significant increase in perforated case numbers during the pandemic despite similar durations of symptoms [25,26,45].

However, numerous authors have documented prolonged times to hospital admission, contrary to previous studies. Ayyildiz et al. aimed to investigate changes in the course of acute appendicitis during COVID-19 and found increased numbers of complicated appendicitis cases and LOSs. Times to hospital admission were significantly extended for the pandemic cohort. During the pre-pandemic period (May 2019 to February 2020), 20.3% of children presented on the first day of symptoms whereas only 2% did so during the pandemic (March 2020 to December 2020). Hospital admission after three or four days from symptom onset more than doubled during the pandemic; this was a statistically important finding [19]. Taşçı et al. found that time from first complaint to hospital admission had a median of 2 days (range 1–8 days) for the pre-pandemic group and 4 days (range 1–8 days) during the pandemic. Overall appendicitis frequency or complicated appendicitis frequency did not change. The authors concluded that delayed hospital admission did not alter outcomes. Another interesting observation was that parental education did not contribute to increased time before hospital admission [28]. Sheath et al. found a similar 2-day delay to presentation with more complicated appendicitis cases [16]. Ergün et al. showed prolonged times between the onset of symptoms and hospital admission, longer hospitalization times before surgery and longer LOSs. Perforation rates increased by 18–19% on average during the pandemic in comparison to the previous three years [41]. Analogous results were also reported by another three authors [49,104,105]. Li et al. observed protracted times from symptom onset to presentation during the pandemic (2.42 ± 2.5 vs. 1.87 ± 1.2 days), and more perforated cases (47% vs. 32%), but less time to incision [17]. Lower median times from ED to incision for children with appendicitis in both 2019 and 2020 were also documented by Sullivan et al. [6]. Delayed presentations from symptom onset by 12 and 24 h with no differences in outcomes have been demonstrated [30,48]. Delgado et al. recorded a significantly longer time to admission in their COVID-19 group, higher rates of complicated appendicitis and higher postoperative complication rates [15,18]. A median delay of one day being associated with worse outcomes was registered by Gerall et al. [37]. A systematic review and meta-analysis including some of the previously mentioned studies, conducted by Köhler et al., demonstrated a symptom duration of 32 h (95% CI 19.39–44.61) before seeking medical help during the pre-pandemic period and 51.5 h during the pandemic (95% CI 31.17–74.53) with statistical significance [11].

## 5. Conclusions

Diagnostic pathways for appendicitis, one of the most common pediatric emergencies, display considerable variation worldwide. This review of the published COVID-19 literature endeavors to display the reasons for delays in the first step of this pathway, i.e., the initial presentation of children to healthcare facilities. A key suggestion that must be effectively communicated to parents is the prompt and comprehensive physical examination of their children by an appropriate healthcare professional. Health literacy needs to be promoted by government-endorsed media campaigns and through educational platforms such as state websites. Novel methods such as social media interventions have been effective in this regard [106]. The evaluation of public health interventions by surveys can help estimate their impact [107,108]. Future research on low-income or rural areas and countries ought to be encouraged. Health-system and public policy should be optimized by utilizing the knowledge gained during the COVID-19 pandemic. Eliminating bureaucracy, and making campaign revisions as needed using feedback, leads to the faster and better implementation of public health interventions, as shown by the US Department of Health and Human Services [109]. The successful application of novel technologies such as telemedicine can be refined by more research whilst having a low threshold for formal patient evaluation where indicated. Maintaining diagnostic and treatment pathways for emergency surgical conditions in the event of a new pandemic, such as pediatric appendicitis, is of great importance.

## Figures and Tables

**Table 1:** Publication details of included studies.

	Author	Type of Study	Study Period	Year	Journal	Country
1	Köhler et al. [11]	Systematic Review and Meta-analysis	February 2021 (date of search)	2021	*Int. J. Surg*.; 95:106148	Germany
2	Delgado-Miguel et al. [15]	Retrospective Cohort	January 2017–March 2020 (C) * March–December 2020	2021	*J. Healthc. Qual. Res.*; 37(4):225–230	Spain
3	Sheath et al. [16]	Retrospective Cohort	March–June 2019 (C) March–June 2020	2021	*J. Paediatr. Child Health*; 57(7):986–989	United Kingdom (UK)
4	Li et al. [17]	Retrospective Cohort	1 March–30 June 2019 (C) 1 March–30 June 2020	2022	*J. Pediatr. Surg.*; 57(5):861–865	Canada
5	Delgado-Miguel et al. [18]	Retrospective Cohort	9 March–13 April 2015–2019 (C) 9 March–13 April 2020	2022	*Eur. J. Pediatr. Surg.*; 32(3):268–273	Spain
6	Ayyıldız et al. [19]	Retrospective Cohort	1 May 2019–29 February 2020 (C) 1 March–31 December 2020	2022	*Ulus Travma Acil Cerrahi Derg*; 28(12):1674–1681	Turkey
7	ANZSCRAFT Collaborative [20]	Retrospective Cohort	20 March–30 April 2018, 2019 (C) 20 March–30 April 2020	2022	*ANZ J. Surg.*; 92(4):736–741	Australia, New Zealand
8	Nassiri et al. [21]	Retrospective Cohort	23 March–31 August 2019 (C) 23 March–31 August 2020	2022	*J. Am. Coll. Emerg. Physicians Open*; 3(2):e12722	United States of America (USA)
9	Toro Rodríguez et al. [22]	Retrospective Cohort	January 2019–February 2020 (C) March 2020–December 2020	2022	*Cir. Pediatr.*; 35(3):131–134	Spain
10	Sullivan et al. [6]	Retrospective Cohort	18 March–31 May 2019 (C) 18 March–31 May 2020	2022	*J. Surg. Res.*; 279:299–303	USA
11	Motazedian et al. [23]	Systematic Review and Meta-analysis	1995–December 2019 (C) December 2019–January 2021	2021	*Arch. Acad. Emerg. Med*.; 10(1):e3	Iran
12	La Pergola et al. [24]	Retrospective Cohort	20 February–20 April 2017–2019 (C) 20 February–20 April 2020	2020	*Front. Pediatr.*; 8:600320	Italy
13	Gaitero Tristán et al. [25]	Retrospective Cohort	January–September 2019 (C) 21 March 2020–6 May 2020	2021	*Pediatr. Emerg. Care*; 37(3):185–190	Spain
14	Esparaz et al. [26]	Retrospective Cohort	October 2019–February 2020 (C) March–May 2020	2021	*J. Surg. Res.*; 268:263–266	USA
15	Ali et al. [7]	Retrospective Cohort	March–May 2019 (C) March–May 2020	2020	*J. Ayub Med. Coll. Abbottabad*; 32(Suppl 1)(4):S621–S624	Pakistan
16	Quaglietta et al. [27]	Retrospective Cohort	February 2018–June 2019 (C) February 2020–June 2021	2023	*J. Pediatr. Surg.*; 58(5):931–938	Canada
17	Taşçı et al. [28]	Retrospective Cohort	20 December 2019–10 March 2020 (C) 11 March 2020–1 June 2020	2022	*Ulus Travma Acil Cerrahi Derg*; 28(8):1095–1099	Turkey
18	Horst et al. [29]	Retrospective Cohort	1 March–31 May 2019 (C) 1 March–31 May 2020	2021	*Pediatr. Radiol.*; 51(11):1991–1999	USA
19	Head et al. [30]	Retrospective Cohort	December 2019–15 March 2020 (C) 15 March 2020–June 2020	2021	*Am. Surg.*; 26:31348211067995	USA
20	Snapiri et al. [13]	Case Series/Retrospective Cohort	1 March–30 April 2019 (C) 1 March–30 April 2020	2020	*Acta Paediatr.*; 109(8):1672–1676	Israel
21	Moratilla Lapeña et al. [31]	Retrospective Cohort	14 March–20 April 2019 (C) 14 March–20 April 2020	2021	*Cir. Pediatr.*; 34(2):85–89	Spain
22	Iantorno et al. [32]	Retrospective Cohort	1 April 2020–31 March 2021	2023	*Surgery*; 173(4):936–943	USA
23	Schäfer et al. [33]	Retrospective Cohort	20 March–31 May 2018, 2019 (C) 20 March–31 May 2020	2021	*Front. Pediatr*.; 9:683607	Germany
24	Yock-Corrales et al. [34]	Prospective Multinational Multicenter Cohort	1 July–11 August 2020	2021	*Pediatr. Infect. Dis. J.*; 40(10):e364–e369	Peru, Costa Rica, Argentina, Colombia, Mexico
25	Tan et al. [35]	Prospective Comparative Cohort	April 2020–January 2022	2022	*Pediatr. Surg. Int*.; 39(1):60	Singapore
26	Rethi et al. [8]	Retrospective Cohort	1 March–30 November 2019 (C) 1 March–30 November 2019	2022	*J. Emerg. Med.*; 63(6):723–728	USA
27	Theodorou et al. [36]	Retrospective Cohort	19 March–19 September 2019 (C) 19 March–19 September 2020	2021	*J. Surg. Res.*; 267:132–142	USA
28	Gerall et al. [37]	Retrospective Cohort	1 March–31 May 2019 (C) 1 March–31 May 2020	2021	*J. Pediatr. Surg*.; 56(5):905–910	USA
29	Farooq et al. [38]	Case-Control Study	April–September 2019 (C) April–September 2020	2021	*BMJ Paediatr. Open*; 5(1):e001066	Bangladesh
30	Pawelczyk et al. [39]	Retrospective Cohort	1 January–31 December 2019 (C) 1 January–December 2020	2021	*Sci. Rep*.; 11(1):23999	Poland
31	Percul et al. [40]	Retrospective Cohort	20 March–20 August 2019 (C) 20 March–20 August 2020	2021	*Arch Argent Pediatr.*; 119(4):224–229	Argentina
32	Ergün et al. [41]	Retrospective Cohort	11 March–30 September 2017–2019 (C) 11 March–30 September 2020	2021	*Turk. J. Surg*.; 37(4):318–323	Turkey
33	Yap et al. [42]	Prospective Comparative Study	1 May 2020–31 January 2021	2023	*J. Pediatr. Surg.*: S0022–3468(23)00172–0	Singapore
34	Bethell et al. [43]	Prospective Multicenter Observational Cohort	13 March–18 June 2017 (C) 1 April–31 July 2020	2022	*J. Pediatr. Surg.*; 57(10):380–385	UK, Ireland
35	Vansevičienė et al. [44]	Retrospective Cohort	16 March–16 June 2019 (C) 16 March–16 June 2020	2021	*Medicina (Kaunas)*; 57(11):1234	Lithuania
36	Patel et al. [45]	Retrospective Cohort	3 March–30 June 2019 (C) 3 March–30 June 2020	2021	*Ann. Med. Surg. (Lond.)*; 71:102901	UK
37	Montalva et al. [46]	Retrospective Cohort	20 January–17 March 2020 (C) 17 March 2020–11 May 2020	2020	*Pediatr. Surg. Int*.; 36(12):1397–1405	France
38	Öztaş et al. [47]	Retrospective Cohort	March 2019–February 2020 (C) March 2020–February 2021	2023	*Ann. Pediatr. Surg*.; 19(1):3	Turkey
39	Dass et al. [48]	Prospective Cohort	1 April–August 2019 (C) 1 April–31 August 2020	2023	*Afr. J. Paediatr. Surg*.; 20(1):40–45	UK
40	Gürünlüoglu et al. [49]	Prospective Cohort	December 2018–May 2021	2023	*Afr. J. Paediatr. Surg*.; 20(2):130–137	Turkey
41	Del Giorgio et al. [50]	Retrospective Cohort	March 2016–March 2020 (C) April 2020–March 2021	2023	*World J. Pediatr.*; 19(3):288–292	Canada
42	Matava et al. [51]	Retrospective, International Multicenter Cohort	April–May 2019 (C) April–May 2020	2023	*Anesthesiology*; Online ahead of print	Canada, Australia, USA

* (C) denotes the control group period.

**Table 2:** Reasons for delayed diagnosis of pediatric appendicitis during COVID-19 pandemic.

1	Public health and social measures
2	Fear of exposure by parents/caregivers/patients/medical staff
3	Telemedicine use
4	COVID-19-positive children with suspected appendicitis
5	Recurrent appendicitis after non-operative management
6	Delayed primary operative management

## Data Availability

Data sharing is not applicable to this article.

## Appendix A

(“paediatrics”[All Fields] OR “pediatrics”[MeSH Terms] OR “pediatrics”[All Fields] OR “paediatric”[All Fields] OR “pediatric”[All Fields] OR “children”[All Fields] OR “children’s”[All Fields]) AND (“appendicitis”[MeSH Terms] OR “appendicitis”[All Fields] OR (“acute”[All Fields] AND “appendicitis”[All Fields]) OR “acute appendicitis”[All Fields]) AND (“COVID-19”[All Fields] OR “COVID-19”[MeSH Terms] OR “COVID-19 vaccines”[All Fields] OR “COVID-19 vaccines”[MeSH Terms] OR “COVID-19 serotherapy”[All Fields] OR “COVID-19 nucleic acid testing”[All Fields] OR “COVID-19 nucleic acid testing”[MeSH Terms] OR “COVID-19 serological testing”[All Fields] OR “COVID-19 serological testing”[MeSH Terms] OR “COVID-19 testing”[All Fields] OR “COVID-19 testing”[MeSH Terms] OR “SARS-CoV-2”[All Fields] OR “SARS-CoV-2”[MeSH Terms] OR “severe acute respiratory syndrome coronavirus 2”[All Fields] OR “ncov”[All Fields] OR “SARS-CoV-19”[All Fields] OR “2019 ncov”[All Fields] OR ((“coronavirus”[MeSH Terms] OR “coronavirus”[All Fields] OR “cov”[All Fields]) AND 2019/11/01:3000/12/31[Date—Publication])) AND (“time”[MeSH Terms] OR “time”[All Fields] OR “concurrent”[All Fields] OR “delay”[All Fields] OR “delayed”[All Fields] OR “delaying”[All Fields] OR “delays”[All Fields] OR “admission”[All Fields]).
